# Phase angle assessed by bioelectrical impedance analysis in patients with colorectal cancer: a systematic review

**DOI:** 10.3389/fonc.2026.1768933

**Published:** 2026-09-30

**Authors:** Yuzhou Mei, Tengfei Wang, Songhao Liu, Haoyu Wang, Zhenguo Han

**Affiliations:** 1 Department of General Surgery, Yuncheng Tongde Hospital, Yuncheng, Shanxi, China; 2 Department of General Surgery, Third Hospital of Shanxi Medical University, Shanxi Bethune Hospital, Shanxi Academy of Medical Sciences, Tongji Shanxi Hospital, Taiyuan, China

**Keywords:** BIA, colorectal cancer, nutritional status, phase angle, prognosis, sarcopenia

## Abstract

**Background:**

Malnutrition, altered body composition, and sarcopenia are common among patients with colorectal cancer (CRC) and are associated with poor treatment tolerance and adverse clinical outcomes. PhA, derived from BIA, reflects cellular membrane integrity and body cell mass and has been increasingly investigated as a potential marker of nutritional status and prognosis in oncology. However, the clinical relevance of PhA in patients with CRC has not yet been systematically synthesized.

**Methods:**

This systematic review was conducted in accordance with the PRISMA 2020 guidelines. PubMed, Embase, Web of Science, and Scopus were searched for observational studies published between January 2000 and October 2025 that assessed PhA in adult patients with CRC. Data extraction and risk-of-bias assessment using the AXIS tool were performed independently by two reviewers. Due to substantial heterogeneity in study design, outcome definitions, and PhA cut-off values, a narrative synthesis was conducted.

**Results:**

A total of 10 studies involving 1,439 patients were included. Lower PhA values were consistently associated with adverse clinical outcomes. Four studies reported significantly shorter overall or progression-free survival among patients with low PhA. Four studies demonstrated associations between low PhA and unfavorable short-term outcomes, including postoperative complications, prolonged hospital stay, cancer-related complications, and early chemotherapy-related toxicity. Several studies also reported positive associations between PhA and indicators of nutritional status, body composition, muscle mass, muscle strength, and hydration balance. One longitudinal study showed that postoperative increases in PhA were accompanied by improvements in body composition parameters and reduced early chemotherapy-related toxicity.

**Conclusions:**

Current evidence indicates that BIA-derived phase angle is associated with nutritional status, body composition, treatment tolerance, and survival outcomes in patients with colorectal cancer. However, heterogeneity in measurement protocols and cut-off values limits direct clinical application. Standardized assessment methods and prospective studies are needed to establish CRC-specific reference values and to determine whether interventions that improve PhA can translate into improved clinical outcomes.

## 1 Introduction

Colorectal cancer (CRC) is the third most commonly diagnosed malignancy worldwide. In 2022, approximately 18.74 million new cancer cases and 9.67 million deaths were reported globally, with CRC accounting for 1.92 million new cases (10.2%) and 904,000 deaths (9.3%), making it the third most frequent cancer and the second leading cause of cancer mortality (1). Incidence continues to rise in many developed regions, while developing countries are experiencing increasing rates due to lifestyle westernization, rising obesity, and dietary transitions (2).

CRC arises from multifactorial causes. Beyond genetic susceptibility and family history, lifestyle-related risk factors—including high-fat and low-fiber diets, excessive intake of red and processed meats, obesity, physical inactivity, smoking, and heavy alcohol consumption—significantly contribute to disease development (3–9). Chronic inflammatory bowel diseases, such as ulcerative colitis and Crohn’s disease, also markedly increase CRC risk (10).

CRC and its treatments frequently impair gastrointestinal function, leading to anorexia, nausea, vomiting, diarrhea, abdominal distension, and bowel obstruction, all of which reduce nutrient intake and absorption (11, 12). Tumor burden and anticancer therapies further exacerbate nutritional decline (13, 14). Consequently, CRC patients are highly susceptible to fatigue, unintended weight loss, malnutrition, and cancer-related cachexia (15– 16). Malnutrition affects 20%–80% of cancer patients and is associated with poor prognosis, reduced treatment response, diminished tolerance to therapy, lower quality of life, and higher healthcare costs (17–24). Cachexia develops in a substantial proportion of CRC patients, with incidences of 50.7% in advanced disease and 29.82% after curative resection (18, 25). Sarcopenia occurs in 12%–60% of CRC patients and is linked to postoperative complications, chemotherapy toxicity, reduced treatment tolerance, and increased mortality (26–29).

Assessment of body composition is therefore essential in evaluating cancer patients, as it provides insight into nutritional status, functional reserve, and prognosis (30). BIA offers a simple, non-invasive method to estimate body composition based on tissue electrical properties. By measuring resistance (R) and reactance (Xc), BIA reflects changes in cellular integrity and hydration status (31–33).

Phase angle (PhA), derived from arctan(Xc/R), serves as an indicator of cellular health, body cell mass, and fluid distribution (34). Typical values range from 5° to 7° in healthy individuals, with lower values suggesting impaired cellular function and age-related decline (35). Low PhA is associated with increased morbidity and mortality across several cancers, including breast, pancreatic, and lung malignancies (35–37). Although emerging evidence indicates prognostic value of PhA in CRC (34), current findings have not been systematically synthesized or critically evaluated.

Despite growing interest in PhA across oncology, no systematic review has specifically synthesized its clinical relevance in colorectal cancer. This review therefore aims to clarify the associations between PhA and nutritional status, body composition, functional capacity, treatment outcomes, and survival in CRC patients.

## 2 Methods

### 2.1 Study design and literature search strategy

This systematic review was conducted using a predefined protocol and reported in accordance with the Preferred Reporting Items for Systematic Reviews and Meta-Analyses (PRISMA) 2020 Statement (38).

The databases, search concepts, eligibility criteria, outcomes of interest, and planned synthesis approach were defined before the formal literature search and study-selection process.

A comprehensive literature search was conducted in PubMed, Embase, Web of Science, and Scopus to identify eligible studies published between January 2000 and October 2025. Because “phase angle” is not a Medical Subject Heading (MeSH) term, it was searched primarily as a free-text phrase. Free-text terms related to phase angle and bioelectrical impedance were combined, where appropriate, with controlled vocabulary for colorectal neoplasms and related concepts. The search syntax was adapted to the indexing system and field structure of each database.

The principal search concepts were phase angle, bioelectrical impedance, and colorectal cancer. Representative free-text terms included “phase angle,” “bioelectrical phase angle”, “bioimpedance phase angle”,”bioelectrical impedance”, “bioimpedance” and “BIA” combined with “colorectal cancer”, “colorectal carcinoma”,”colorectal neoplasm”, “colon cancer” and “rectal cancer”. In PubMed, relevant MeSH terms, including “Colorectal Neoplasms”,”Colonic Neoplasms”, “Rectal Neoplasms” and “Electric Impedance” were used where appropriate. Corresponding controlled vocabulary was applied in Embase.

Boolean operators, phrase searching, truncation, and database-specific field tags were used as appropriate. No study-design filter was applied in order to maximize search sensitivity. The reference lists of included studies and relevant reviews were also manually screened to identify additional eligible records. Reference management and duplicate removal were performed using Zotero and EndNote X9.

### 2.2 Protocol registration

This systematic review was not prospectively registered in PROSPERO because registration was not completed before the formal literature search and study-selection process began. Nevertheless, a review protocol was developed before study selection and predefined the research question, eligibility criteria, databases and search strategy, outcomes of interest, data-extraction procedures, risk-of-bias assessment, and synthesis approach. All stages of the review, including literature searching, study selection, data extraction, risk-of-bias assessment, evidence synthesis, and reporting, were conducted using these predefined methods and reported in accordance with the PRISMA 2020 Statement. No major deviations from the predefined protocol occurred during the review process.

### 2.3 Inclusion and exclusion criteria

We included original studies if they met the following criteria: ① phase angle (PhA) assessed using BIA or bioelectrical impedance vector analysis (BIVA); ② adult participants≥18 years; ③ studies reporting at least one association between PhA and a clinically relevant outcome, such as nutritional status, body composition, functional or inflammatory markers, treatment-related complications, or survival; ④ articles published in English. The exclusion criteria were: ① did not involve cancer populations or did not include colorectal cancer as the primary study population; ② review, conference and symposium articles; ③ full text was not available.

### 2.4 Data extraction and analysis

Full texts of all eligible studies were examined in detail. Two reviewers independently extracted all data using a standardized Excel form, and discrepancies were resolved by discussion. Extracted information included study characteristics (first author, year, country, study design, sample size, age and sex distribution), tumor-related variables, and details of PhA assessment (BIA/BIVA methods and measurement protocols).

Owing to substantial heterogeneity in study design, patient populations, phase angle measurement protocols, cut-off values, effect measures, and outcome definitions, quantitative meta-analysis was considered inappropriate. Therefore, a structured narrative synthesis was conducted with reference to the Synthesis Without Meta-analysis (SWiM) reporting guideline. Studies were grouped according to clinically relevant outcome domains, including: (1) general and cancer-related characteristics; (2) body composition, muscle function, and physical performance; (3) nutritional status or nutritional risk; (4) short-term clinical outcomes; and (5) survival outcomes.

Within each outcome domain, study characteristics, direction and magnitude of associations, statistical significance, adjustment for potential confounders, and risk of bias were compared across studies. Because the included studies reported heterogeneous effect measures, including correlation coefficients, odds ratios, hazard ratios, group comparisons, and survival estimates, these results were not converted into a common standardized effect measure. Conclusions were based on the consistency, direction, and strength of the reported associations, together with study design, sample size, adjustment for confounding factors, and methodological quality. No formal vote counting based solely on statistical significance was performed.

### 2.5 Narrative synthesis

The narrative synthesis was structured with reference to the SWiM reporting guideline. Eligible studies were grouped according to prespecified clinically relevant outcome domains rather than according to the statistical significance of their findings. These domains included body composition and muscle function, nutritional status, short-term clinical outcomes, and survival outcomes.

For each domain, we summarized the study population, PhA assessment method, reported effect measure, direction and magnitude of association, statistical uncertainty, adjustment for confounding factors, and risk of bias. Owing to substantial heterogeneity in outcomes and statistical measures, no common effect metric was calculated. The overall interpretation was based on the consistency and direction of findings, the magnitude and precision of reported associations, and the methodological limitations of the contributing studies.

### 2.6 Risk of bias assessment

Risk of bias was assessed using the AXIS tool, which was specifically developed for observational studies, including cross-sectional studies. The AXIS tool was selected because the majority of included studies were observational in design, and it provides a structured evaluation of study objectives, methodological quality, sample selection, measurement validity, management of confounding factors, and reporting transparency.

Compared with tools designed primarily for randomized controlled trials or cohort studies with intervention comparisons, AXIS is more suitable for evaluating heterogeneous observational studies without requiring a predefined intervention-control framework. Therefore, it was considered appropriate for the present review, in which most studies investigated associations between PhA and clinical outcomes rather than treatment effects.

However, the AXIS tool also has limitations. It does not provide an overall numerical quality score and does not generate a quantitative estimate of bias magnitude. In addition, it cannot fully address residual confounding or establish causal relationships in observational studies. Therefore, AXIS assessments were not used as exclusion criteria but were incorporated into the interpretation of findings. Studies with important methodological limitations, such as inadequate adjustment for confounders, unclear PhA measurement protocols, or small sample sizes, were interpreted cautiously when evaluating the consistency and strength of associations.

### 2.7 Certainty of the evidence

A formal assessment of the certainty of evidence using the Grading of Recommendations Assessment, Development and Evaluation (GRADE) approach was not performed in this review. This decision was based on the substantial heterogeneity among the included studies in terms of study design, patient populations, disease stages, clinical settings, BIA measurement protocols, timing of PhA assessment, phase angle cut-off values, outcome definitions, and statistical effect measures.

The included studies consisted of prospective, retrospective, observational, and cross-sectional designs, with outcomes reported using heterogeneous measures, including correlation coefficients, odds ratios, hazard ratios, survival estimates, and between-group comparisons. Because these findings could not be organized into sufficiently comparable outcome-specific evidence bodies, a formal GRADE assessment was considered unlikely to provide meaningful or reliable certainty ratings.

Instead, the strength and consistency of evidence were interpreted through structured narrative synthesis, considering study design, sample size, consistency of findings, adjustment for potential confounding factors, reported effect estimates, and risk of bias. However, this approach does not replace a formal certainty-of-evidence assessment, and the overall certainty of current evidence should therefore be interpreted cautiously.

## 3 Results

### 3.1 Study selection

A total of 162 records were identified through systematic database searching, including 26 from PubMed, 39 from Embase, 36 from Scopus, and 61 from Web of Science. After removal of duplicates (n = 64), 98 records remained for screening, of which 88 were excluded for not meeting the inclusion criteria. Consequently, 10 studies were included in this systematic review (**Figure 1**).

**Figure 1:**
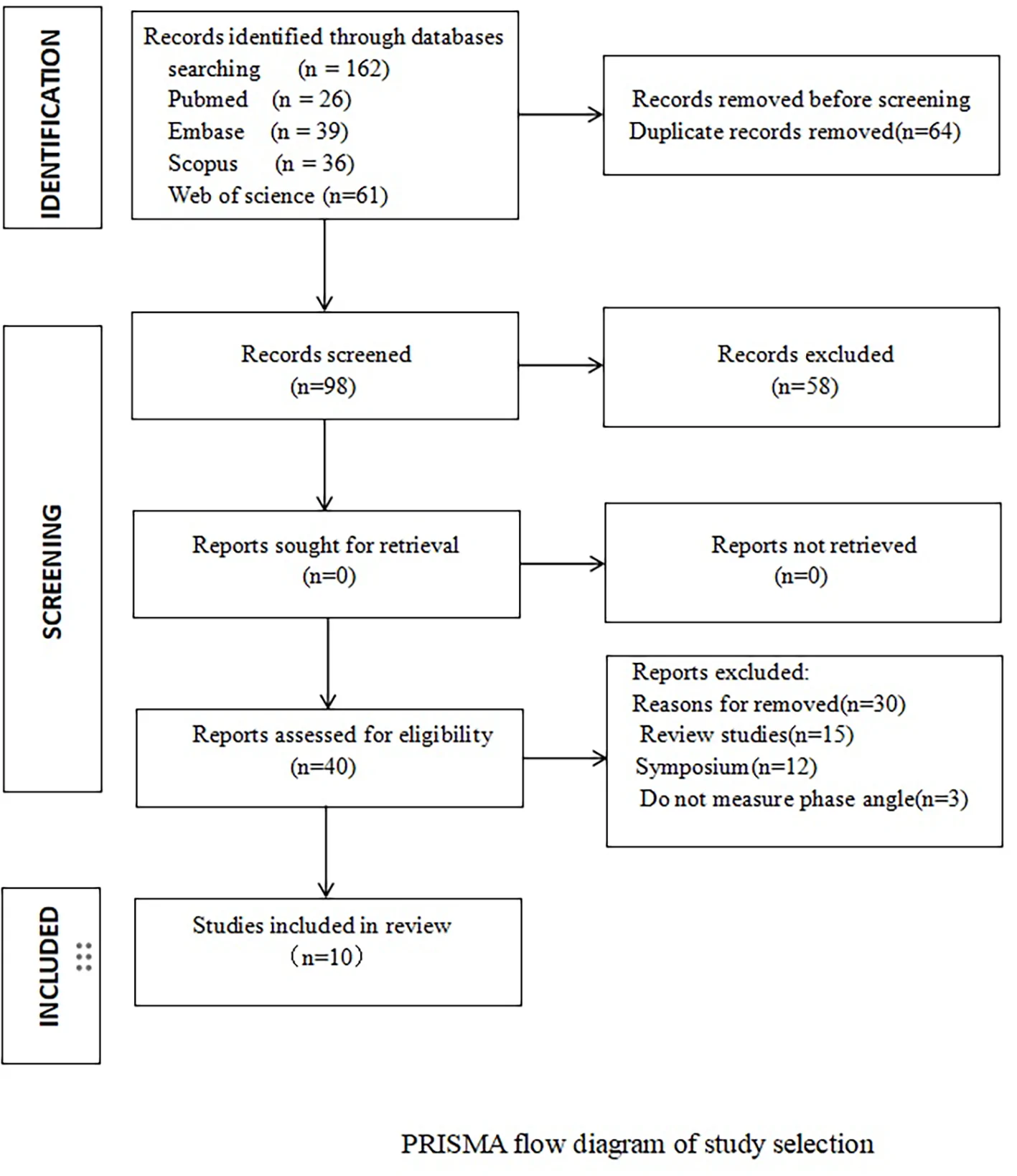
PRISMA flow diagram of study selection.

Among the included studies, two were conducted in the United States and two in Brazil, while one study each was conducted in South Korea, China, Spain, Slovenia, Croatia, and Egypt. The included articles were published between 2000 and 2025, and all studies evaluated both male and female participants.

### 3.2 Characteristics of included studies

The 10 included studies were published between 2004 and 2025 and represented diverse geographic regions. Study designs included 4 prospective studies (39–42), 1 observational study (43), 2 retrospective cohort studies (34, 44), and 3 cross-sectional analyses (45–47). Sample sizes ranged from 52 to 250 participants. Most studies focused exclusively on colorectal cancer (CRC) patients, while one study included mixed gastrointestinal malignancies. The main characteristics of the included studies are summarized in **Table 1**. A more detailed description of study characteristics and additional variables is provided in **Supplementary Table 1**.

**Table 1:** General characteristics of included studies.

Study	Aim	Design
Gupta et al,2004 (37)	Evaluate the prognostic value of PhA in advanced CRC	Retrospective
Gupta et al,2008 (45)	Assess the association between SGA and PhA in advanced CRC	Cross-sectional
Han et al, 2022 (43)	Evaluate longitudinal changes in PhA during chemotherapy in postoperative	Observational
Souza et al, 2021 (46)	Investigate associations between PhA and muscle mass, density, and function	Cross-sectional
Liu et al, 2024 (44)	Evaluate PhA and risk of postoperative complications in CRC Retrospective	Retrospective
Vegas-Aguilar et al, 2023 (47)	Assess predictive value of PhA for complications and sarcopenia risk in CRC	Cross-sectional
Gulin Jet al,2023 (39)	Determine whether PhA predicts postoperative complications in gastrointestinal cancer	Prospective
Barao et al,2017 (40)	Examine associations between PhA, nutritional status, and survival in elderly CRC	Prospective
Kekez D et al, 2025 (41)	Assess relationship between PhA and survival outcomes in metastatic CRC	Prospective
Mohamed Sad et al,2020 (42)	Evaluate associations between PhA and OS/PFS in metastatic CRC	Prospective

PhA, Phase Angle; CRC, colorectal cancer.

For narrative synthesis, the included studies were grouped into four principal outcome domains: body composition and physical function, nutritional status, short-term clinical outcomes, and survival outcomes. Some studies contributed evidence to more than one domain. Because of differences in study populations, PhA thresholds, outcome definitions, and statistical measures, the findings were summarized using study-level effect estimates and their direction, rather than being combined into a single quantitative estimate. The main associations between PhA and the clinical variables reported across the included studies are summarized in **Table 2**.

**Table 2:** Main associations between PhA and variables indicated in the studies.

Study	Tumour characteristic	PhA vs. other variables
Gupta et al, 2004 (37)	Colorectal cancer stage IV	Median survival: PhA ≤ 5.57: 8.6 months (95% CI:4.8–12.4),PhA > 5.57: 40.4 months (95% CI: 21.9–58.8; p < 0.0001)
Gupta et al, 2008 (45)	Colorectal cancer stages III and IV	SGA grade correlated with PhA(r = 0.33, p = 0.004)
Han et al, 2022 (43)	Colorectal cancer stages I–IV	PhA high group SMM, SMI, FFM were higher(p< 0.05); Lower NLR (p = 0.001); Lower early chemotherapy toxicity(30% vs. 64.7%; P = 0.041)
Souza et al, 2021 (46)	Colorectal cancer stages I–IV	SMI(r = 0.70, p < 0.05);SMD(r = 0.47, p < 0.05); HGS(r = 0.50, p < 0.05);GS(r = 0.31, p < 0.05)(r = 0.31, p < 0.05)
Liu et al, 2024 (44)	Colorectal cancer stages I–IV	Postoperative complications (OR = 0.615, 95% CI: 0.405–0.933; p =0.002)
Vegas-Aguilar et al, 2023 (47)	Colorectal cancer stages I–IV	Cancer complications (female, OR = 0.15,95%CI:0.03–0.81;p<0.05); Sarcopenia (male, OR = 0.42, 95% CI:0.19–0.95; p < 0.05)
Gulin J et al, 2023 (39)	Colorectal cancer (91.5%)other cancers (8.5%)	Hospital stay (r = −0.4;P = 0.001);Higher PhA in patients without complications(5.64 ± 0.72 vs 5.09 ± 0.98; p= 0.036)
Barao et al, 2017 (40)	Colorectal cancer stages I–IV	Median survival:PhA ≤ 5:23months(95%CI:18.5–27.9);PhA > 5:46.2 months (95% CI:35.9 –56.5;p < 0.001); mortality risk (HR = 0.456, 95% CI: 0.263 –0.792; p = 0.005).
Kekez D et al, 2025 (41)	Metastatic colorectal cancer	PFS: 14.8 months (high PhA) vs 10.5 months(low PhA), p =0.048;Better disease control:96.3%vs 74.6% (p < 0.001)
Mohamed Sad et al, 2020 (42)	Metastatic colorectal cancer	Low T stage, low fat ratio, high BMI, and better performance status associated with high PhA(PhA ≥ 4. 1) (P < 0.05);Longer OS (P = 0.032)

BMI, body mass index; CRC, colorectal cancer; FFM, fat-free mass; GS, gait speed; HGS, handgrip strength; HR, hazard ratio; NLR, neutrophil-to-lymphocyte ratio; OS, overall survival; PFS, progression-free survival; SGA, Subjective Global Assessment; SMD, skeletal muscle density; SMM, skeletal muscle mass; SMI, skeletal muscle index.

### 3.3 Association between PhA and general or cancer-related characteristics

The included studies covered heterogeneous CRC populations across different disease stages and clinical settings. Five studies enrolled patients with stage I–IV CRC (40, 43, 44, 46, 47), three focused exclusively on metastatic CRC (34, 41, 42), one included patients with stage III–IV locally advanced or metastatic disease (45), and one predominantly included patients undergoing surgery for gastrointestinal malignancies, most of whom had colorectal or related lower gastrointestinal cancers (39). This heterogeneity in disease stage and treatment setting should be considered when interpreting associations between PhA and demographic or tumor-related characteristics.

Evidence regarding the relationship between PhA and age was limited and inconsistent. Han et al. reported significantly higher PhA values in patients younger than 70 years than in those aged 70 years or older (P < 0.001) (43), whereas Mohamed Sad et al. found no significant differences in PhA according to age or sex in a cohort of patients with metastatic CRC (42). These discrepancies may partly reflect differences in disease stage, sample size, and patient characteristics.

Evidence concerning tumor burden and functional status was more limited. In the metastatic CRC cohort reported by Mohamed Sad et al., lower PhA values were more frequent in patients with T4 disease than in those with T2–T3 tumors (P = 0.004), while higher PhA values were observed in patients with better ECOG performance status (42). However, these findings were derived from a single study and were not consistently confirmed in multivariable analyses.

Overall, the available evidence suggests that lower PhA may be associated with older age, greater tumor burden, and poorer functional status in some CRC populations. Nevertheless, the small number of studies, inconsistency in age-related findings, heterogeneity of disease stage, and limited adjustment for confounding factors preclude firm conclusions regarding whether PhA is independently associated with demographic or tumor-related characteristics.

### 3.4 Association between PhA and body composition, muscle function, and physical performance

Four studies evaluated the relationship between phase angle and body composition, muscle-related parameters, or physical performance (42, 43, 46, 47). Across these studies, higher PhA was generally associated with more favorable muscle quantity, muscle quality, physical function, and hydration-related characteristics, although the strength and type of association varied according to the assessed outcome and study design.

The strongest reported association was observed between PhA and computed tomography–derived skeletal muscle index, with Souza et al. reporting a correlation coefficient of r=0.70 (46). PhA was also positively associated with skeletal muscle density (r=0.47), handgrip strength (r=0.54), and gait speed (r=0.31), suggesting that PhA may reflect not only muscle quantity but also muscle quality and functional performance (46). Consistent with these findings, Vegas-Aguilar et al. reported positive associations between PhA and multiple indicators of lean tissue and cellular mass, including fat-free mass, appendicular skeletal muscle mass, skeletal muscle mass, skeletal muscle index, body cell mass, and related indices (47). In contrast, PhA was negatively associated with extracellular water-related ratios, including ECW/TBW, ECW/ICW, and ECW/BCM, indicating that lower PhA may also reflect less favorable fluid distribution and cellular hydration (47).

Sex-stratified findings suggested that the physiological correlates of PhA may differ between men and women. In women, PhA appeared to be more closely related to nutritional status, hydration balance, and fat distribution, whereas in men, stronger associations were observed with skeletal muscle mass, muscle dimensions, and handgrip strength (47). However, these sex-specific findings were exploratory and were derived from subgroup analyses; therefore, they require confirmation in larger prospective cohorts.

Mohamed Sad et al. also found that patients with higher PhA had greater body weight, BMI, and muscle mass, together with a lower body fat percentage (42). However, this study primarily reported between-group comparisons and did not provide correlation coefficients or consistently adjusted estimates. Consequently, the independent relationship between PhA and these body composition variables remains uncertain.

Longitudinal evidence was available from only one study. Han et al. reported that patients whose PhA increased after surgery had higher skeletal muscle mass, skeletal muscle index, and fat-free mass index than those whose PhA decreased (43). Improvements in PhA were also accompanied by lower inflammatory marker levels and a lower incidence of early chemotherapy-related toxicity (43). These findings suggest that serial changes in PhA may provide information on postoperative recovery and treatment tolerance beyond that obtained from a single baseline measurement. Nevertheless, because the evidence was derived from one observational cohort, causal relationships cannot be established.

Overall, the available evidence was directionally consistent in showing that higher PhA is associated with greater muscle mass, better muscle function, higher body cell mass, and more favorable hydration status. However, most studies were cross-sectional or based on unadjusted analyses, and substantial heterogeneity existed in body composition methods, PhA assessment protocols, and statistical approaches. Therefore, PhA should currently be interpreted as a complementary indicator of muscle and cellular health rather than as an independent measure of sarcopenia or physical function.

### 3.5 Association between PhA and nutritional status or nutritional risk

Three studies examined the association between phase angle and nutritional status or nutrition-related indicators (40, 43, 45). Overall, the findings consistently suggested that lower PhA was associated with poorer nutritional status, although the evidence was based on different assessment methods and outcome measures.

Gupta et al. reported a modest positive correlation between PhA and Subjective Global Assessment classification (r=0.33, P = 0.004), and well-nourished patients had significantly higher PhA values than malnourished patients (45). The corresponding AUC of 0.70 indicated moderate discriminatory ability, suggesting that PhA may contribute to nutritional assessment but is unlikely to replace established tools such as SGA.

Barao et al. found that both severe malnutrition assessed by the Patient-Generated Subjective Global Assessment and low PhA were independently associated with increased mortality (40). However, because the direct relationship between PG-SGA and PhA was not examined, these findings suggest parallel prognostic relevance rather than equivalence between the two measures.

Han et al. further reported that longitudinal increases in PhA were accompanied by higher albumin levels and lower inflammatory marker levels (43). This finding suggests that changes in PhA may reflect concurrent improvements in nutritional and inflammatory status, although the contribution of hydration status and disease recovery cannot be excluded.

Taken together, the available evidence suggests that PhA may provide complementary information on nutritional and physiological vulnerability in patients with colorectal cancer. However, the small number of studies, differences in nutritional assessment methods, and limited direct comparisons with established tools reduce the certainty of the evidence. PhA should therefore be regarded as a complementary marker rather than a standalone measure of malnutrition.

### 3.6 Association between PhA and short-term clinical outcomes

Four studies evaluated short-term clinical outcomes, including postoperative complications, hospital length of stay, cancer-related complications, and chemotherapy-related toxicity (39, 43, 44, 47). Overall, the findings were broadly consistent in showing that lower PhA was associated with a higher risk of unfavorable short-term outcomes.

The strongest evidence was derived from the study by Liu et al., in which PhA remained independently associated with postoperative complications after logistic regression analysis. Each one-unit increase in PhA was associated with an approximately 38% reduction in complication risk (OR = 0.615, 95% CI: 0.405–0.933, P = 0.002) (44). This finding was supported by Gulin et al., who reported significantly lower PhA values in patients who developed postoperative complications and a moderate inverse correlation between PhA and hospital length of stay (r = −0.40, P = 0.001) (39).

Vegas-Aguilar et al. also found that higher PhA was associated with a lower risk of cancer-related complications in female patients (OR = 0.15, 95% CI: 0.03–0.81) (47). However, this result was derived from a sex-specific subgroup analysis and should therefore be interpreted cautiously.

Evidence regarding chemotherapy-related toxicity was less consistent. Han et al. reported a lower incidence of early toxicity in patients whose PhA increased after surgery than in those whose PhA decreased (30.0% vs. 64.7%, P = 0.041), whereas no significant difference was observed for overall chemotherapy toxicity (43). This suggests that dynamic changes in PhA may be more closely related to early postoperative recovery and initial treatment tolerance than to cumulative toxicity.

Taken together, the available evidence supports a probable association between lower PhA and adverse short-term clinical outcomes. Nevertheless, differences in outcome definitions, timing of PhA measurement, subgroup analyses, and adjustment for confounding factors limit direct comparison across studies. PhA should therefore be considered a complementary risk-stratification marker rather than an independent predictor for all short-term outcomes.

### 3.7 Association between PhA and survival outcomes

Four studies evaluated the association between phase angle and survival outcomes in patients with colorectal cancer (34, 40–42). Despite differences in patient populations, disease stages, and PhA cut-off values, all studies reported associations in the same direction, with lower PhA corresponding to poorer overall survival or progression-free survival.

Two studies reported substantial differences in median overall survival between low- and high-PhA groups. Gupta et al. found that patients with PhA ≤5.57 had a markedly shorter median overall survival than those with higher PhA values (8.6 vs. 40.4 months, P<0.001P<0.001P<0.001) (34). Similarly, Barao et al. reported shorter median survival in patients with PhA ≤5 than in those with PhA >5 (23.2 vs. 46.2 months, P<0.001P<0.001P<0.001) (40).

Importantly, the association between PhA and survival remained significant after multivariable adjustment in two studies. Barao et al. found that PhA remained independently associated with mortality after adjustment for age, tumor stage, ECOG performance status, and PG-SGA score (HR = 0.456, 95% CI: 0.263–0.792, P = 0.005P=0.005P=0.005) (40). In metastatic CRC, Kekez et al. reported that higher PhA was independently associated with longer progression-free survival (HR = 0.64, 95% CI: 0.42–0.98), together with a higher disease control rate (41). Mohamed Sad et al. also observed improved overall survival in patients with PhA ≥4.1, although detailed median survival estimates were not reported (42).

Taken together, the direction of the survival association was consistent across studies, and adjusted analyses suggest that the prognostic relevance of PhA may not be fully explained by disease stage, performance status, or nutritional status. However, the reported PhA thresholds varied from approximately 4.1 to 5.57, and the included populations differed substantially in age, disease stage, and treatment setting. Therefore, although PhA may serve as a complementary prognostic marker, the available evidence does not support a universal CRC-specific cut-off value or a precise pooled estimate of prognostic effect.

### 3.8 Risk of bias results

According to the AXIS assessment, most included studies demonstrated a moderate risk of bias. The most common methodological limitations included single-center design, lack of sample size justification, limited adjustment for potential confounding factors, and incomplete reporting of non-responders or follow-up details. Nevertheless, most studies clearly defined their objectives, applied standardized phase angle measurement procedures, and used appropriate statistical analyses.

## 4 Discussion

### 4.1 Principal findings and biological interpretation

This systematic review found that lower phase angle was generally associated with poorer nutritional status, unfavorable body composition and muscle-related parameters, a higher risk of postoperative or treatment-related adverse outcomes, and worse survival in patients with colorectal cancer (34, 39–47). These findings suggest that PhA may reflect a dimension of physiological vulnerability that is not fully captured by conventional anthropometric or biochemical indicators.

The biological relevance of PhA is related to its dependence on tissue resistance and reactance. PhA is influenced by body cell mass, cell membrane integrity, muscle tissue, and intracellular–extracellular water distribution (31, 32). A lower PhA may therefore reflect reduced metabolically active tissue, muscle depletion, impaired cellular function, systemic inflammation, and extracellular fluid expansion. Similar associations between lower PhA and adverse nutritional, functional, postoperative, and survival outcomes have also been reported in patients with liver disease, gastric cancer, hematological malignancies, lung cancer, gynecological cancer, breast cancer, head and neck cancer, and other clinically vulnerable populations (48–58).

Previous studies have also linked lower PhA with sarcopenia and reduced body cell mass (59, 60). However, accurate interpretation requires consideration of hydration status and body composition, because similar PhA values may arise from different combinations of resistance and reactance (61). Bioelectrical impedance vector analysis may provide additional information by helping distinguish changes in cellular mass from alterations in hydration (62). Therefore, PhA should be interpreted as an integrative marker of cellular and physiological vulnerability rather than as a specific diagnostic measure of malnutrition or sarcopenia.

Cancer cachexia may provide a plausible biological link between CRC progression and lower PhA. Cachexia is characterized by systemic inflammation, reduced dietary intake, skeletal muscle and adipose tissue loss, and alterations in body-fluid distribution (63–65). These changes may reduce metabolically active body cell mass, impair cell membrane integrity, and increase the relative extracellular water compartment. Because PhA is influenced by body cell mass, membrane function, and intracellular–extracellular water distribution, these cachexia-related physiological changes may contribute to lower PhA values (32, 63–65). In patients with CRC, cachexia may also occur during chemotherapy and further contribute to progressive nutritional deterioration and loss of body composition (66)

Gut microbiota dysbiosis may indirectly contribute to this pathway in CRC by impairing intestinal barrier function, promoting systemic inflammation, and reducing nutrient digestion or absorption (65, 67–69). These processes may aggravate muscle catabolism, nutritional deterioration, and fluid imbalance, which could subsequently be reflected by a lower PhA. However, none of the studies included in this review directly examined the association between gut microbiota composition and PhA. Therefore, the proposed microbiota–cachexia–PhA pathway should be regarded as biologically plausible but currently unconfirmed rather than as an established mechanism. Nevertheless, the associated nutritional deterioration, muscle loss, and systemic inflammation remain clinically relevant because they may contribute to poorer treatment tolerance and adverse outcomes in patients with CRC (70, 71).

Importantly, the relationship between PhA and clinical outcomes should not be interpreted as a direct causal relationship. A low PhA may represent the cumulative effect of multiple pathological processes, including inflammation, muscle depletion, altered hydration, and reduced functional reserve.

### 4.2 CRC-specific features, methodological heterogeneity, and comparison with other malignancies

Several characteristics may make PhA particularly relevant in CRC. Patients frequently experience reduced oral intake, bowel obstruction, diarrhea, gastrointestinal bleeding, altered bowel function, and treatment-related gastrointestinal symptoms. Colorectal surgery may further induce acute inflammatory stress, muscle catabolism, extracellular fluid shifts, and temporary functional decline. These CRC-specific factors may explain why PhA was associated not only with survival but also with nutritional status, body composition, postoperative complications, hospital stay, and early chemotherapy-related toxicity (39–44, 46, 47).

The sequential treatment pathway of CRC may also increase the potential value of longitudinal PhA assessment. Measurements performed at diagnosis, before surgery, at discharge, during chemotherapy, or after treatment may reflect different biological states. A preoperative measurement may primarily reflect baseline nutritional and functional reserve, whereas a postoperative or treatment-period measurement may additionally reflect inflammation, intravenous fluid administration, tissue injury, treatment toxicity, and recovery.

Interpretation is further complicated by methodological heterogeneity across the included studies. Differences existed in BIA device type, operating frequency, electrode configuration, fasting status, recent physical activity, food and alcohol intake, hydration status, edema, diuretic use, time of day, patient position, measurement timing, reassessment frequency, and follow-up procedures. These factors can influence tissue impedance and fluid distribution and may therefore alter PhA independently of nutritional or clinical status (72–74). Therefore, although the direction of associations was relatively consistent, the current evidence does not support the application of a universal PhA threshold for CRC patients.

Despite these methodological differences, the findings of the present review are broadly consistent with previous systematic-review evidence from other malignant tumors. A systematic review in breast cancer reported that lower PhA was generally associated with poorer nutritional and prognostic outcomes, while also highlighting substantial heterogeneity in study populations, measurement protocols, and thresholds (36). In addition, previous studies have demonstrated that cancer treatment may be accompanied by substantial alterations in body composition, which should be considered when interpreting nutritional and functional indicators in oncology populations (75). Similarly, Arab et al. reported that lower PhA was associated with poorer survival across adult cancer populations (76). However, differences in tumor biology, disease stage, treatment pathways, gastrointestinal symptoms, and perioperative fluid changes limit direct extrapolation of findings from other malignancies to CRC.

Nevertheless, CRC may represent a unique clinical setting for PhA assessment because nutritional decline and fluid imbalance can be influenced not only by systemic cancer-related factors but also by gastrointestinal dysfunction, bowel-related symptoms, and surgical interventions. Therefore, CRC-specific validation is necessary before applying PhA thresholds established in other malignancies.

The methodological strength of the included CRC studies also varied. Findings derived from adjusted longitudinal or cohort analyses provide stronger evidence than unadjusted cross-sectional correlations, simple between-group comparisons, or exploratory subgroup analyses. Some studies showed that PhA remained associated with complications or survival after adjustment for relevant clinical factors (40, 41, 44), whereas others did not consistently control for age, sex, disease stage, inflammation, hydration status, comorbidities, or treatment exposure (39, 42, 43, 45–47). The apparent consistency of the findings should therefore not be interpreted as indicating equivalent evidential strength across all studies.

### 4.3 Clinical implications

From a clinical perspective, PhA may be incorporated into a multimodal nutritional and functional assessment rather than used as an isolated decision-making tool. A low or decreasing PhA could prompt further evaluation using established instruments such as NRS-2002, SGA, or PG-SGA, together with assessment of recent weight loss, dietary intake, skeletal muscle mass, handgrip strength, inflammatory status, comorbidities, and hydration.

Before colorectal surgery, low PhA may help identify physiologically vulnerable patients who warrant more detailed nutritional and functional evaluation. These patients may be considered for individualized prehabilitation, including nutritional counseling, adequate energy and protein provision, exercise-based interventions when feasible, and correction of dehydration, fluid overload, anemia, or metabolic abnormalities.

Postoperatively, persistently low or decreasing PhA may support closer surveillance of oral intake, fluid balance, infectious complications, functional recovery, prolonged hospitalization, and tolerance of adjuvant treatment. Serial measurements may be particularly informative when interpreted together with clinical findings, laboratory parameters, muscle-related measures, and established nutritional tools.

The 2025 ESPEN guideline on clinical nutrition in surgery highlights the importance of comprehensive nutritional and functional assessment, including emerging indicators such as PhA and handgrip strength, for identifying patients at increased risk of postoperative complications (77). This is consistent with the present review, in which lower PhA was associated with postoperative complications and prolonged hospital stay (39, 44, 47). Nevertheless, current evidence does not establish a universal CRC-specific PhA threshold for initiating nutritional intervention, modifying oncological treatment, or determining surveillance intensity. PhA should therefore complement rather than replace established clinical, nutritional, and functional assessment methods.

### 4.4 Future research directions

Future studies should use adequately powered, multicenter prospective designs and adopt standardized BIA protocols. Device model, operating frequency, electrode configuration, fasting status, bladder emptying, recent physical activity, hydration requirements, body position, time of day, and timing relative to surgery or systemic treatment should be clearly specified.

Longitudinal studies are needed to determine whether serial changes in PhA provide greater prognostic value than a single baseline measurement. Repeated measurements may help distinguish transient perioperative fluid shifts from persistent deterioration in nutritional status, muscle mass, cellular health, or functional recovery.

Future analyses should consistently adjust for important confounding factors, including age, sex, disease stage, systemic inflammation, hydration status, comorbidities, and treatment exposure. Reporting age- and sex-adjusted standardized PhA values in addition to raw values may also improve comparability across populations.

Interventional studies should determine whether nutritional support, exercise-based prehabilitation, postoperative rehabilitation, or optimized fluid management can improve PhA and whether such changes translate into fewer complications, better treatment tolerance, improved quality of life, or longer survival. Future trials should also clarify whether PhA is merely a marker of disease severity or whether improvement in PhA reflects effective recovery interventions.

Overall, current evidence supports PhA as a promising complementary marker for assessing physiological vulnerability in CRC patients. However, the available evidence remains insufficient to establish PhA as an independent prognostic biomarker or a treatment-guiding parameter. Future standardized prospective studies are required to determine its incremental clinical value beyond established nutritional, functional, and oncological factors.

## 5 Limitations

To the best of our knowledge, this is the most up-to-date review focusing specifically on BIA-derived PhA in patients with colorectal cancer. However, several limitations must be acknowledged. Despite conducting a comprehensive search across multiple databases (including engines beyond PubMed), only ten studies met the inclusion criteria, and among these, nine specifically analyzed PhA in CRC populations. Notably, although four relevant studies were published after 2022, the remaining articles were published between 2004 and 2021, indicating a relative paucity of recent evidence. The review was not prospectively registered in PROSPERO, which may reduce transparency regarding the original review plan. However, a predefined protocol was developed before study selection, and no major deviations from the planned methods occurred.

Several included studies did not clearly report the exact timing of PhA measurements, and different bioelectrical impedance devices were used across studies, which may influence PhA values and limit the comparability and generalizability of proposed cut-off points. A key future objective will be to standardize BIA protocols and improve the comparability and performance of different analyzers. In addition, PhA measurements are sensitive to multiple physiological and behavioral factors, including recent vigorous physical activity, food and alcohol intake, hydration status (dehydration or edema), use of diuretics, and menstrual cycle phase, all of which may affect the results (72). Diurnal variation in total body water and fluid distribution can also influence PhA; therefore, it is recommended that measurements be performed at a consistent time of day to enhance reliability (73). Patient positioning (standing vs. supine) has a substantial impact on PhA, as gravitational effects on fluid distribution result in significantly lower values in the standing position compared with the supine position (74). These sources of variability highlight the need for strict standardization of measurement conditions.

The risk-of-bias findings should also be considered when interpreting the results. Although the AXIS assessment suggested generally acceptable methodological quality among the included studies, most available evidence was derived from observational studies. Therefore, potential selection bias, residual confounding, and measurement bias cannot be completely excluded. In this review, risk-of-bias assessments were considered together with study design, sample size, adjustment for confounding factors, and consistency of findings when interpreting the clinical relevance of PhA. Consequently, the reported associations should be interpreted cautiously, particularly when considering the potential clinical application of PhA.

Given these methodological inconsistencies, a wide range of PhA cut-off values has been reported in the literature. As such, the thresholds summarized in this review should be interpreted with caution, and further research is needed to establish robust, population- and disease-specific reference values for CRC patients.

Although the narrative synthesis was structured with reference to the SWiM reporting guideline, full compliance with all SWiM items was not feasible. The SWiM guideline was primarily developed for systematic reviews of intervention effects, whereas the present review included heterogeneous observational studies examining multiple nutritional, functional, clinical, and prognostic outcomes. In addition, the included studies reported diverse effect measures, including correlation coefficients, odds ratios, hazard ratios, survival estimates, and between-group comparisons, which could not be reliably converted into a common effect metric. Consequently, the synthesis relied partly on qualitative comparisons of the direction, magnitude, and consistency of the reported associations. This approach may be susceptible to subjective interpretation, selective emphasis on statistically significant findings, and unequal weighting of studies with different sample sizes and methodological quality. Therefore, the apparent consistency of findings should not be interpreted as equivalent to a pooled quantitative effect, and the certainty and precision of the conclusions remain limited. These limitations may have resulted in either overestimation or underestimation of the clinical relevance of PhA.

In addition, because the formula for calculating PhA is arctan(Xc/R) × (180/π), different positions on the R–Xc graph may theoretically yield identical PhA values. Lukaski and colleagues demonstrated that patients with malnutrition or dehydration may exhibit PhA values comparable to those of healthy individuals, despite having reduced body cell mass and altered hydration status (32). Similarly, overweight individuals may present PhA values similar to those of muscular athletes, despite substantial differences in body composition. These observations highlight a fundamental limitation of PhA: while it reflects the ratio of reactance to resistance, it does not fully differentiate the underlying physiological contributors. Therefore, when using PhA to evaluate nutritional status or cellular health, it should always be interpreted in conjunction with complementary indicators—such as body composition parameters, hydration assessments, or BIVA-derived vector analysis—to improve diagnostic accuracy and clinical relevance.

## 6 Conclusion

The current scientific literature on the use of BIA-derived phase angle (PhA) in patients with colorectal cancer remains limited. Nevertheless, early identification of nutritional deterioration following diagnosis and throughout anticancer treatment is essential for maintaining functional status, preventing malnutrition and sarcopenia, and ultimately improving quality of life. This systematic review summarizes the available clinical evidence regarding the use of PhA in CRC patients. Most studies demonstrate that PhA effectively reflects cellular membrane integrity, body cell mass, and body composition, and has been associated with nutritional status, sarcopenia risk, inflammatory burden, treatment tolerance, postoperative complications, and survival outcomes. Lower PhA values generally indicate poorer nutritional and functional status, heightened systemic inflammation, greater vulnerability to short-term adverse events, and worse long-term survival.

Although the number of studies is small and measurement methods lack standardization, PhA shows promising potential as a simple, non-invasive, and clinically meaningful prognostic indicator. Future longitudinal and prospective studies are needed to clarify the dynamic relationship between PhA and changes in nutritional status among CRC patients, to evaluate the impact of various interventions (such as resistance training and nutritional support) on PhA, and to determine the clinical significance of PhA changes over time. Such evidence will contribute to the development of individualized nutritional and rehabilitation strategies for patients with colorectal cancer. PhA may serve as a practical tool for risk stratification and nutritional assessment in colorectal cancer patients at the population level.

## Data Availability

The original contributions presented in the study are included in the article/**Supplementary Material**. Further inquiries can be directed to the corresponding author.
