# Supplementary material for: Phase angle assessed by bioelectrical impedance analysis in patients with colorectal cancer: a systematic review
Source: Front Oncol. 2026 Sep 30;16:1768933. doi: 10.3389/fonc.2026.1768933 (PMC13661394; doi:10.3389/fonc.2026.1768933)
Supplement: Supplementary file 1 [file Table1.docx]

# **TableS1.**General characteristics of included studies

| **Study** | **Aim** | **Design** | **N** | **Sex** | **Age** | **BIA Device** | **Electrode distribution** | **Vector** |
| --- | --- | --- | --- | --- | --- | --- | --- | --- |
| Gupta et al,2004[35] | Evaluate the prognostic value of PhA in advanced CRC | Retrospective | 52 | both | 55.8±10.8 | BIA-101Q analyzer(RJL Systems, MI, USA) | Whole body | No |
| Gupta et al,2008[39] | Assess the association between SGA and PhA in advanced CRC | Cross-sectional | 73 | both | 56±11.4 | BIA-101Q analyzer(RJL Systems, MI, USA) | Whole body | No |
| Han et al, 2022[40] | Evaluate longitudinal changes in PhA during chemotherapy in postoperative | Observational | 160 | both | 63.0±10.7 | InBody 770 (InBody Co, Seoul, Korea) | NS | No |
| Souza et al, 2021[41] | Investigate associations between PhA and muscle mass, density, and function | Cross-sectional | 190 | both | 60.5±11.3 | Quantum II（RJL Systems, Detroit, MI, USA） | Whole body | No |
| Liu et al, 2024[42] | Evaluate PhA and risk of postoperative complications in CRC Retrospective | Cross-sectional | 190 | both | 62.1±12.5 | NS | NS | No |
| Vegas-Aguilar et al,2023[43] | Assess predictive value of PhA for complications and sarcopenia risk in CRC | Cross-sectional | 127 | both | 66.5±8.9 | Nutrilab bioimpedance transducer (Akern, Florence, Italy) | Whole body | No |
| Gulin J et  al,2023[44] | Determine whether PhA predicts postoperative complications in gastrointestinal cancer | Prospective | 70 | both | 65.0±10.7 | Bodystat Quadscan 4000 (Douglas, UK) | Whole body | No |
| Barao et  al,2017[45] | Examine associations between PhA,nutritional status, and survival in  elderly CRC Prospective | Prospective | 250 | both | 70.9±7.49 | QuadScan 4000 (Bodystat Ltd, Isle of Man, UK) | Whole body | No |
| Kekez D et al,2025[46] | Assess relationship between PhA and survival outcomes in metastatic | Prospective | 238 | both | Median (62) | Tanita MC-780MA-N(Tanita Corp., Tokyo, Japan) | Whole body | No |
| Mohamed Sad et al, 2020[47] | Evaluate associations between PhA and OS/PFS in metastatic CRC | Prospective | 89 | both | Median (40) | TANITA MC-780U analyzer | Whole body | No |

Age (years): Mean ± Standard Deviation;PhA Phase Angle;CRC colorectal cancer;NS Not Specified
