# Supplementary material for: Phase angle assessed by bioelectrical impedance analysis in patients with colorectal cancer: a systematic review
Source: Front Oncol. 2026 Sep 30;16:1768933. doi: 10.3389/fonc.2026.1768933 (PMC13661394; doi:10.3389/fonc.2026.1768933)
Supplement: Supplementary file 2 [file Table2.docx]

# **TableS2.**Main associations between PhA and variables indicated in the studies

| **Study** | **Tumour characteristics** | **PhA vs Age and/or gender** | **PhA vs other variables** | **Statistical method** |
| --- | --- | --- | --- | --- |
| Gupta et al,2004[35] | Colorectal cancer stage IV | NI | Median survival:PhA ≤ 5.57: 8.6 months (95% CI: 4.8–12.4), PhA > 5.57: 40.4 months (95% CI: 21.9–58.8; p < 0.0001) | Univariate Kaplan–Meier survival analysis |
| Gupta et al,2008[39] | Colorectal cancer stages III and IV | NI | SGA grade correlated with PhA(r = 0.33, p = 0.004) | Spearman correlation |
| Han et al,2022[40] | Colorectal cancer stages I–IV | Higher in patients  <70 years  (p < 0.001) | PhA high group SMM, SMI, FFM were higher(p< 0.05);Lower NLR (p = 0.001); Lower early chemotherapy toxicity(30% vs. 64.7%; P = 0.041) | Mann–Whitney U test  Independent t-test  Chi-square test |
| Souza et al,2021[41] | Colorectal cancer stages I–IV | NI | SMI（r = 0.70, p < 0.05）;SMD（r = 0.47, p < 0.05）;HGS（r = 0.50, p < 0.05）;GS（r = 0.31, p < 0.05） | Pearson correlation  Spearman correlation |
| Liu et al,2024[42] | Colorectal cancer stages I–IV | NI | Postoperative complications (OR = 0.615, 95% CI: 0.405–0.933; p = 0.002) | Multivariable logistic regression |
| Vegas-Aguilar et al, 2023[43] | Colorectal cancer stages I–IV | NI | Cancer complications (female, OR = 0.15, 95% CI = 0.03–0.81;p< 0.05); Sarcopenia (male, OR = 0.42, 95% CI = 0.19–0.95; p < 0.05) | Multivariable logistic regression |
| Gulin J et al,2023[44] | Colorectal cancer (91.5%, unspecified stages)； other cancers (8.5%) | NI | Hospital stay (r = −0.4;P = 0.001);Higher PhA in patients without complications(5.64 ± 0.72 vs 5.09 ± 0.98; p = 0.036） | Pearson correlation  Independent t-test |
| Barao et al,2017[45] | Colorectal cancer stages I–IV | NI | MMedian survival:PhA ≤ 5: 23.2 months (95% CI:18.5–27.9) PhA > 5:46.2 months (95% CI:35.9–56.5;p < 0.001);mortality risk (HR = 0.456, 95% CI: 0.263–0.792; p = 0.005). | Kaplan-Meier  Cox proportional hazards model |
| Kekez D et al,2025[46] | Metastatic colorectal cancer | NI | PFS: 14.8 months (high PhA) vs 10.5 months (low PhA), p = 0.048; Better disease control: 96.3% vs 74.6% (p < 0.001) | Kaplan-Meier  Chi-square test |
| Mohamed Sad et al,2020[47] | Metastatic colorectal cancer | No difference by age/sex | Low T stage, low fat ratio, high BMI,and better performance status associated with high PhA (PhA ≥ 4.1) (P < 0.05); Longer OS (P = 0.032) | Chi-square test  Univariate Kaplan–Meier  survival analysis |

**Abbreviations:** BMI, body mass index; CRC, colorectal cancer; FFM, fat-free mass; GS, gait speed; HGS, handgrip strength; HR, hazard ratio; NLR, neutrophil-to-lymphocyte ratio; NI, not investigated;

OS, overall survival; PFS, progression-free survival; SGA, Subjective Global Assessment; SMD, skeletal muscle density; SMM, skeletal muscle mass; SMI, skeletal muscle index.
